# GENAVi: a shiny web application for gene expression normalization, analysis and visualization

**DOI:** 10.1186/s12864-019-6073-7

**Published:** 2019-10-16

**Authors:** Alberto Luiz P. Reyes, Tiago C. Silva, Simon G. Coetzee, Jasmine T. Plummer, Brian D. Davis, Stephanie Chen, Dennis J. Hazelett, Kate Lawrenson, Benjamin P. Berman, Simon A. Gayther, Michelle R. Jones

**Affiliations:** 1 Center for Bioinformatics and Functional Genomics, Department of Biomedical Science, Cedars-Sinai Medical Center, Los Angeles, CA 90048 USA; 2 Women’s Cancer Program, Samuel Oschin Comprehensive Cancer Institute, Cedars-Sinai Medical Center, Los Angeles, CA 90048 USA

**Keywords:** Next generation sequencing, RNA-seq, Shiny, GUI, Differential expression, Visualization, Normalization

## Abstract

**Background:**

The development of next generation sequencing (NGS) methods led to a rapid rise in the generation of large genomic datasets, but the development of user-friendly tools to analyze and visualize these datasets has not developed at the same pace. This presents a two-fold challenge to biologists; the expertise to select an appropriate data analysis pipeline, and the need for bioinformatics or programming skills to apply this pipeline. The development of graphical user interface (GUI) applications hosted on web-based servers such as Shiny can make complex workflows accessible across operating systems and internet browsers to those without programming knowledge.

**Results:**

We have developed GENAVi (Gene Expression Normalization Analysis and Visualization) to provide a user-friendly interface for normalization and differential expression analysis (DEA) of human or mouse feature count level RNA-Seq data. GENAVi is a GUI based tool that combines Bioconductor packages in a format for scientists without bioinformatics expertise. We provide a panel of 20 cell lines commonly used for the study of breast and ovarian cancer within GENAVi as a foundation for users to bring their own data to the application. Users can visualize expression across samples, cluster samples based on gene expression or correlation, calculate and plot the results of principal components analysis, perform DEA and gene set enrichment and produce plots for each of these analyses. To allow scalability for large datasets we have provided local install via three methods. We improve on available tools by offering a range of normalization methods and a simple to use interface that provides clear and complete session reporting and for reproducible analysis.

**Conclusion:**

The development of tools using a GUI makes them practical and accessible to scientists without bioinformatics expertise, or access to a data analyst with relevant skills. While several GUI based tools are currently available for RNA-Seq analysis we improve on these existing tools. This user-friendly application provides a convenient platform for the normalization, analysis and visualization of gene expression data for scientists without bioinformatics expertise.

**Electronic supplementary material:**

The online version of this article (10.1186/s12864-019-6073-7) contains supplementary material, which is available to authorized users.

## Background

The rapid decrease in cost and increased access to the tools needed to generate RNA-Seq data have resulted in it being widely incorporated into basic science research [1]. However, tools for those without bioinformatics expertise to analyze the data have not been developed at the same pace, and analysis of RNA-Seq data generally requires some bioinformatics, programming and expert knowledge, or the purchase of commercially available software [2]. Providing user-friendly methods for the normalization, analysis and visualization of gene expression data can enhance the incorporation of high throughput genomics into basic science research by those without necessary expertise. We identified three key features of a user-friendly RNA-Seq analysis application and designed an open source application to address these needs;

- tools should be stable and hosted online, independent of operating system or internet browser and not depend on local installation;
- high quality analysis tools should be packaged in a way that does not require expert knowledge of programming (such as use of R) but be accessed via a graphical user interface (GUI);
- the output and results should be downloadable in an easy to use format for data tables and plots.

The development of analytic tools for RNA-Seq data has grown considerably, and selecting the correct processing pipeline and normalization strategy has a significant impact on downstream analysis [3]. It is common for the initial steps of data processing (quality control, alignment and feature identification and counting) to be performed by a core bioinformatics service, often available from core labs that generate RNA-Seq data at a relatively low cost. The downstream analysis (normalization, differential expression and plotting the results of each) often requires several iterations and can be more efficiently performed by the researcher who designed the experiment, if they have the analytic expertise. This scenario presents a two-fold challenge to biologists; selection of the most appropriate data analysis pipeline, which often consists of multiple independent analytic packages [4], and the need for sufficient bioinformatics skills to apply this pipeline to their processed RNA-Seq data.

The development of GUI based software tools can address these challenges by collecting different software packages within one platform and making complex analytical tools accessible to scientists without bioinformatics expertise, or access to a data analyst with relevant skills. Some GUI based applications are currently available for RNA-Seq analysis [5–10], however we have developed GENAVi to improve on these existing tools. GENAVi provides improvements over previously published tools in five key areas. We have provided flexible hosting options that allow users to install locally should they have the expertise, as well as web hosting that provides flexible access across browsers. Four data normalization methods are available to users, providing increased flexibility for data analysis. The interactive nature of the plots included in GENAVi allows users to explore the results of their analyses. A broad set of options for pathway enrichment allows users to design an analysis most suited to their own hypothesis and dataset easily and quickly. Finally, the clear, but complete, reporting available on each of the analysis tabs allows users to easily record their analysis settings and results in a single report, supporting reproducible and transparent analysis. We have used R and Bioconductor packages that allow the application to be easily maintained and updated as analytic methods continue to mature for the normalization and analysis of gene expression data, and by hosting our software as a Shiny web app the tool is functional across all computing platforms and operating systems.

### Implementation

#### Hosting/capacity

GENAVi is a Shiny web app [11] built in an R framework [12] that provides four types of data normalization, four types of data visualization, differential expression analysis (DEA) and gene set enrichment analysis using count level RNA-Seq data. GENAVi is available in three formats: as a hosted web application that runs within an internet browser, as a local installation of a Shiny app that runs from within R, and as a docker image that can be downloaded and run from within the container. GENAVi is hosted on the Shiny server, and can most easily be accessed via web browser and the url https://junkdnalab.shinyapps.io/GENAVi/ [13]. This hosted application can process a counts matrix of 100 samples (with ~ 40 M reads per sample, assigned as counts to more than 58,000 genes/features) through normalization and DEA in 22 min of compute time. Experiments including more than one hundred samples should be run on a local machine with more than 8GB of RAM to accommodate rlog normalization and perform DEA. GENAVi can be installed and run locally by executing the following command in R [shiny::runGitHub (“GENAVi”, “alpreyes”)]. Alternatively, users can build GENAVi from this Docker image file that provides the complete R and Bioconductor environment needed for the app to function [14].

#### Gene expression data

GENAVi can be used to analyze the provided RNA-Seq datasets or users can upload their own mouse or human RNA-seq data for normalization and analysis. The application currently provides a panel of cell lines that are commonly used as models for ovarian and breast cancer in our research program (listed in Additional file 1: Table S1 and Additional file 2: Table S2, GEO (GSE114332)). RNA-Seq was performed according to methods described in Additional file 4: Text 1. To assess the quality of our cell line RNA-Seq, we used a custom quality control script that calls FastQC (version 0.11.5) and fastqscreen (version 0.6.3) to provide input for MultiQC (version 1.3). Forty two cell lines from the Cancer Cell Line Encyclopedia (CCLE) related to ovarian cancer were collected as .bam files and converted to fastq using Picard SamToFastq. After review of QC metrics we aligned each read pair using a custom STAR script (version 2.5.1b) to Gencode v26 (hg38 build of the reference human genome). This process produced a bam file for each sample which we then used for quantification of gene-level expression. We next used the featureCounts function of the subread package (version 1.5.2) to count the number of reads that mapped to a reference gene [15]. The featureCounts function counts reads against a genomic feature (exons, transcripts or genes) rapidly by applying chromosome hashing and feature blocking, and can be used for both single and paired end sequencing [15]. Our use of the featureCounts function along with the Gencode v26 annotation file accounted for alternate transcripts of each gene and collapsed those transcripts to gene level. The complete code for these scripts is available as an installable package at https://github.com/alpreyes/GENAVi.

We maintained two separate matrices for our in house generated RNA-Seq and those from the CCLE. Due to differences in library preparation (non-stranded vs. stranded) that introduce batch effects that cannot be addressed with normalization, data from the CCLE and our in-house RNA-Seq should not be directly compared. A feature counts matrix and metadata file are available for CCLE ovarian cancer cell lines as Additional file 5: Table S4 and Additional file 6: Table S5. The resulting data table for the panel of 20 cell lines we generated (Additional file 2: Table S2) had information on the quantified expression of 58,219 features across our 20 cell lines and also included gene identification information, chromosomal position, strand information, and gene length. This data frame along with its corresponding metadata table (Additional file 3: Table S3) served as the foundation for assembling GENAVi.

Users can bring their own human or mouse RNA-Seq datasets to the application in the form of a counts matrix. A matrix of genes as rows and samples as columns can be uploaded as a .csv file. We have deposited example files (featureCounts matrix for newly generated data and gene count matrix for TCGA breast cancer samples, with their MetaData) in a publicly available Google Drive folder (as detailed in the User Guide, Additional file 4: Text 2) and supplied them as Supplemental tables, with detailed instructions on their format and how to upload them to the app provided within the vignette and tutorial. All user data is uploaded temporarily, and the data is cleared from the server at the end of each session. A search bar allows query of the displayed data table and clicking on genes individually will select genes for visualization and clustering. A larger number of genes can be selected simultaneously by uploading a list of gene names as a text file, or either directly entering or pasting a list of gene names into the ‘Gene list filter’ text box, and selected genes will move to the top of the displayed data table and only these selected genes will then be displayed in the expression heatmap and cluster plots.

#### Normalization of expression data

**Fig. 1:**
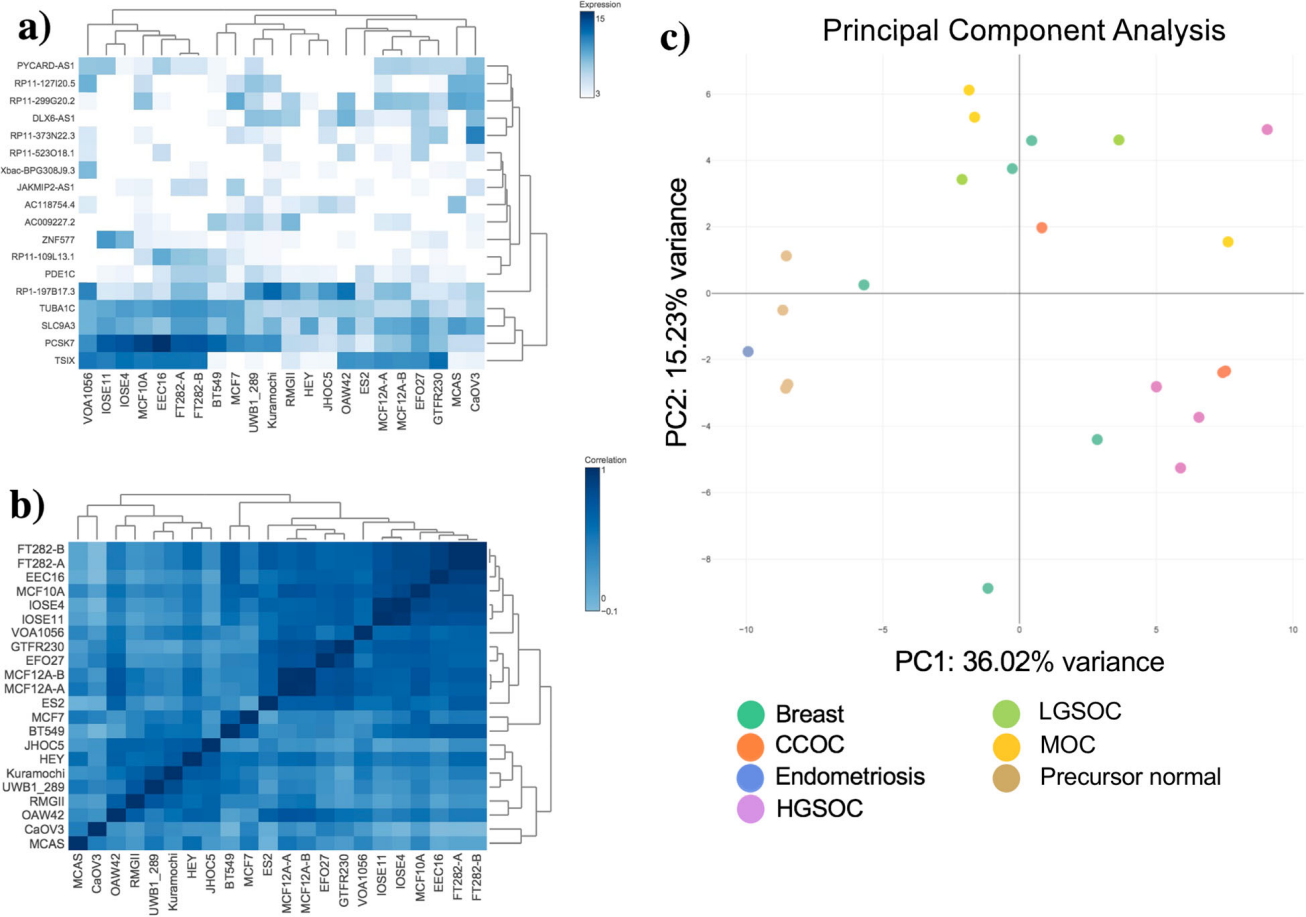
GENAVi provides a GUI for gene expression normalization and differential expression analysis. **a**) Expression Heatmap of differentially expressed genes in ovarian cancer cell lines. **b**) Clustered heatmap identifies similarities in samples based on selected genes. Normal precursor cells are clustered separately from cancer cell lines. **c**) Principal Component Analysis (PCA) plot shows sample clustering based on selected genes.

Four methods of normalization are available with the Select Transform drop-down menu. The first transformation available is labelled “row normalization” and is based on row normalizing the expression of each individual gene across all samples, resembling a t-score normalization (Fig. 1a). The second transformation, logCPM (counts per million) is implemented from the edgeR package [16]. CPM values are calculated for every feature in every sample, and these CPM values are then log2 transformed. The last two transformations GENAVi utilizes come from the DESeq2 package [17]: the variance stabilizing transformation (vst) and the regularized logarithmic transformation (rlog). The vst method produces transformed expression values similar to those produced by the log2 transform while also using a negative binomial model to account for changes in the variance of genes based on the dynamic range of mean expression. Lastly, the rlog method transforms raw counts based on the number of reads in individual samples (representing noise that can be introduced by differences in sequencing depth between samples). Since this transformation is computationally intensive we limited its use to cases with fewer than 30 samples. We have selected these four methods for data normalization as they represent the most robust and widely applied methods for transforming and normalizing RNA-Seq data for visualization and analysis. We have not included the rank-based normalization method, as recent analyses have found it is not suitable for normalization of large differences across many features. The technical and experimental artifacts that can generate very large differences in gene expression across samples do not perform well under rank-based normalization [18, 19]*.* A thorough review of normalization methods for RNA-Seq data is provided to users in the application Vignette.

#### Visualization of normalized expression data

The second feature of GENAVi is the visualization of gene expression across samples. This is accomplished in four separate outputs within the “Visualization” tab. The first two plots can be viewed under the “Expression plots” subtab. When the user wants to view the expression of a single gene across all samples and selects this gene in the data table, a bar plot is generated in the plotting subtab. When the user has selected more than one gene to view, GENAVi uses the package iheatmapr [20] to generate an interactive heatmap that displays an expression matrix of the user-selected genes across all samples of the data table (Fig. 1a). Under the “Clustering plots” subtab, the user can view an interactive heatmap visualizing the Pearson correlation matrix calculated using either all genes or user-selected genes in the displayed data table (Fig. 1b). Within the clustering heatmap the order of samples along the vertical and horizontal axes are determined by the Pearson distance between samples. To allow users to view the relationships between and across samples with different sets of genes selected we have also provided Principal Components Analysis (PCA) using the base R function prcomp. The user can view either a 2D or 3D PCA plot of the complete or filtered data set, and using labels or groups provided in the metadata for each sample can opt to color points by these labels. This represents an important quality control step in RNA-Seq data analysis, and allows non-expert users to identify sample or group outliers quickly. All of the plots generated can be downloaded as a .png file and have interactive features allowing zoom, data point selection and for data values for points to be displayed when the cursor is hovered over them. These functions allow the user to explore their dataset by searching for genes of interest, including those that are identified in the DEA module of the app and rapidly generate plots.

#### Differential expression analysis

**Fig. 2:**
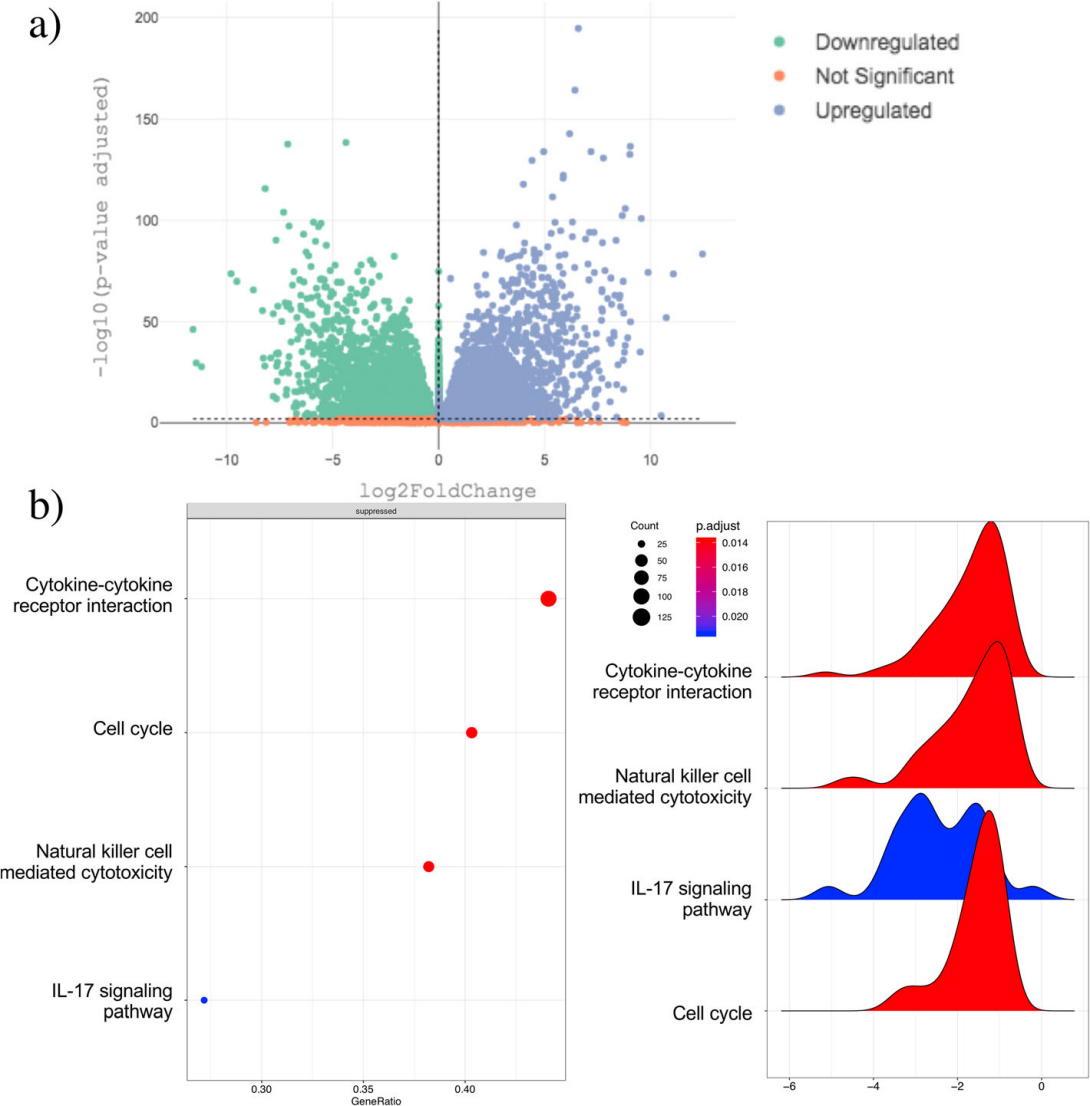
GENAVi offers rapid DEA using DESeq2 and gene set or pathway enrichment analysis for biological interpretation of analysis results. **a**) volcano plot showing results of DEA performed using a subset of TCGA breast cancer cases. **b**) Results of gene set enrichment can be plotted in several ways, including as a dot plot (left) or a ridgeline plot (right) to identify over represented biological pathways in DEA results

GENAVi enables the user to perform and visualize differential expression analysis using the DESeq2 workflow [17]. Under the “Differential Expression Analysis” tab, the user can upload a metadata file which is then used to group samples in the displayed data table based on experimental design and conditions. Next, the user specifies the group to be used as baseline for all differential expression comparisons as well as any covariates in the experimental design provided within the metadata. The result of the DEA is displayed in a table resembling the DESeq2 results table, as well as an interactive volcano plot that can be customized through drop-down menus (Fig. 2a). This module of the application allows complex analysis models to be generated easily in a GUI, providing analytic tools to researchers without R or bioinformatics expertise.

#### Gene set enrichment analysis

Finally, GENAVi allows users to perform gene set enrichment analysis to identify functional profiles of the differentially expressed genes (Fig. 2). A thorough guide to gene pathway analysis describing these tools is available as an online resource from Yu et al. [21]. Under the “Enrichment analysis” tab, the user can upload the DEA analysis results and select between performing an Over Representation Analysis (ORA) [22] or a gene set enrichment analysis (GSEA) [23]. We provide five databases of annotated gene sets that can be used for the enrichment analysis; Molecular Signatures Database - MSigDB [24], WikiPathways [25], Kyoto Encyclopedia of Genes and Genomes (KEGG) [26] and Gene Ontology (GO) [27, 28]. This analysis can be computationally intensive, depending on the file size used as input, however a progress bar allows the user to track the analysis. We have provided an additional dataset of RNA-Seq count level data for breast cancer samples from The Cancer Genome Atlas processed using TCGABiolinks [29] that is suitable for the exploration of DEA and GSEA functionalities provided by GENAVi. Briefly, we select the luminal and basal subtypes from African American patients to generate a modest sized “real world” example of DEA between two groups with appropriate samples as a model for users. The code used within TCGA Biolinks to access and download the count level HTSeq data from TCGA is available at the GENAVi project github repository [30].

#### Reporting and reproducibility of analysis

To provide complete reporting of the session in which analysis is performed a report can be generated on each of the four analysis tabs of the application. This report (generated as an html file) records the analysis performed by reporting the options selected within the R Shiny session. The libraries used and commands required to perform the analysis as selected through the GUI are provided, along with interactive tables and figures that are generated during the analysis. Additionally the report provides the complete list of packages and their versions used within the session to perform the analysis, allowing users to maintain an easy to view and store record of their analysis and results. This option is available via the Generate Report option on each tab, and reports are downloaded.

## Results

We have developed a user-friendly GUI based Shiny web application to host our own catalog of RNA-seq data and provide a platform for those without bioinformatics expertise to analyze their own data. We performed a search for open source RNA-Seq analysis tools that did not require any programming expertise to run (either in R or at the command line), used stable and maintained established packages in R or Bioconductor and that operate through a GUI that are currently active. Six tools satisfied these four criteria; START [5], iDEP [31] [6], DEBrowser [32], DEGUST [8], DEIVA [33], and DEApp [34]. Each of these tools have some similar functionality to one another, and to GENAVi, however GENAVi improves on their functions in a number of ways (Table 1). We split the comparison of functions into four categories: Hosting, User Data, Normalization, and Outputs. Hosting refers to the various platforms from where these applications can be used, User Data refers to the upload and prefiltering of low count features of user data. Normalization refers to both the use of packages as well as the number of normalization available within each app; an “NA” in this category indicates that the application does perform some normalization but does not allow users to toggle between different normalization schema. Outputs refers to the different types of plots and tables produced by each app as well as the nature of these as interactive or downloadable.

**Table 1:** A summary of comparisons between GENAVi and tools with shared functionality

	GENAVi	START	iDEP	DEBrowser	DEGUST	DEIVA	DEApp
Hosting							
GUI	Yes	Yes	Yes	Yes	Yes	Yes	Yes
URL hosted	Yes	Yes	Yes	No	Yes	Yes	Yes
Active	Yes	Yes	Yes	Yes	Yes	Yes	Yes
run from R	Yes	Yes	No	Yes	No	No	Yes
Open source	Yes	Yes	Yes	Yes	Yes	Yes	Yes
Docker	Yes	No	Yes	No	No	No	No
User data							
Upload	Yes	Yes	Yes	Yes	Yes	Yes	Yes
Prefiltering	Yes	No	Yes	Yes	Yes	No	Yes
Bulk Feature Selection	Yes	No	No	No	No	Yes	No
Normalization							
Normalization	Yes	Yes	Yes	Yes	No	No	Yes
Methods Available	4	2	3	3	NA	NA	NA
Visualization							
Interactive expression heatmap	Yes	Yes	Yes	Yes	No	No	No
Interactive correlation heatmap	Yes	No	Yes	Yes	No	No	No
Volcano Plot	Yes	Yes	Yes	Yes	Yes	Yes	No
Interactive PCA plotting	Yes	Yes	Yes	Yes	No	No	Yes
2D and 3D PCA plotting	Yes	No	No	No	No	No	No
DEA							
DEA with packages	Yes	No	Yes	Yes	Yes	No	Yes
Complex model	Yes	Yes	Yes	Yes	No	No	Yes
lfc shrinkage	Yes	No	No	No	No	No	No
Pathway Analysis							
ORA	Yes	No	No	No	No	No	No
GSEA ranking method	Yes	No	Yes	Yes	No	No	No
Enrichment Analysis	Yes	No	Yes	Yes	No	No	No
Reproducible Analysis and Documentation							
Session and code report	Yes	No	Yes	No	Yes	No	No
Complete analysis report	Yes	No	Yes	No	No	No	No

The greatest improvement offered by GENAVi is the flexibility in data normalization approaches available. The use of appropriate normalization prior to data visualization is important for the correct interpretation of RNA-Seq data. We offer four normalization methods, and provide a detailed explanation of each in the application user guide (Additional file 4: Text 2) to assist non-expert users. While four of these tools were developed using the shiny framework, only START and DEApp are hosted at a URL and also offer the possibility to launch from within R locally. This functionality allows GENAVi to be installed and hosted locally with a single line of code, or by a bioinformatician who would like to host GENAVi on a local server for collaborators or customers of a core laboratory who are biologists. GENAVi’s greatest differential in this area is its’ availability as a docker image which makes it the best solution in terms of scalability, as it could be easily deployed on different operating systems for very large projects, since all dependencies are already configured in the docker image. By making GENAVi available in these three forms, we allow our application to have the compute power needed to process user data of varying magnitude.

GENAVi offers the greatest flexibility in data normalization approaches, which we believe to be an important functionality. The use of appropriate normalization prior to data visualization is important for the correct interpretation of RNA-Seq data, and we have addressed this in the application user guide (Additional file 4: Text 2) to assist non-expert users. While the applications listed above perform some normalization of RNA-seq data, few allow users to toggle between different options and view their effects on clustering and gene expression between samples. We believe this to be an important advantage of our tool. While we do relieve the user of some difficult software implementation decisions, the choice of normalization for clustering is dependent on the nature of data uploaded. Therefore the user needs the ability to choose different normalization methods to visualize and analyze their data throughout their workflow. Lastly, GENAVi offers more flexibility for data visualization than other available tools. While similar packages allow the user to search and select individual genes to visualize, they do not provide the subset of most variable genes, or as many options for selecting multiple genes. This offers a significant improvement for the plotting of quality control and analysis results, which may require the selection of hundreds or thousands of genes for visualization by cluster plot. A summary of the relevant functions discussed above as well as other features benchmarked between the tools is shown in Table 1.

To further compare the functions offered in GENAVi with similar tools, we processed the RNA-Seq count level data from the panel of 20 cell lines through each, and benchmarked functions and runtimes for comparable analysis steps such as performing DEA and GSEA. When comparing the DEA functions of each tool we observed that the four tools that perform complex models (including covariates and batch correction) analysis times were closely matched, and took between 1 min 30 s and 2 min 30 s. We also noted that one of the comparable tools, DEIVA [33], does not perform complex DEA analysis, but requires the upload of DESeq2 results and is then able to visualize these results. Upload of our releatively small dataset (20 samples) took approximately 10 min, while upload of externally generated results from the same file to GENAVi required less than a minute. Only two of the comparable tools offer GSEA (iDEP, and DEBrowser), and this process required between 30 s and 1 min 30 s depending on specific parameters used.

In performing a thorough comparison of tools with some shared functionality we observed an additional strength in the design and use of GENAVi; the simple user interface requires a small amount of training despite utilizing complex analysis approaches. Additionally, we noted the advantage of a single upload of count level data and complete analysis pipeline means that GENAVi requires the least amount of processing and formatting of input files. The simple but complete reporting of the code underlying the R session as a single html file within each analysis tab of the application that includes interactive tables and figures also provides a useful improvement over other available tools. It allows users to easily record the analysis settings selected by the user and combines these with the results of this analysis. This report has the added benefit of giving unexperienced users exposure to the code required to perform the analyses they have designed within the GUI, potentially providing them with a starting point to take their analyses directly to R should they wish to extend their analysis with additional Bioconductor packages. While processing our dataset through these six tools, we concluded that our software presented the most accessible user interface by separating analysis steps into distinct tabs. Although other tools separate different plots and outputs, only START, DEBrowser, and DEApp break apart entire analysis steps into distinct locations within their respective GUIs. We believe this to be a major advantage of GENAVi as it intends to facilitate researchers without bioinformatics expertise to utilize an RNA-seq analysis pipeline. A summary of the relevant functions discussed above as well as other features benchmarked between the tools is shown in Table 1.

## Conclusions

We have developed GENAVi as a user-friendly GUI based application to enhance the ability of researchers without bioinformatics expertise to incorporate high throughput RNA-Seq data into their research. By providing four options for data normalization, PCA, DEA and gene set enrichment paired with a range of visualization of RNA-Seq data options our goal is to enable researchers to perform their own data analysis and visualization. Additionally, we have offered a variety of ways to host GENAVi that will allow more expert users to scale our tool to meet the needs of their own RNA-seq analysis should they have very large experiments or wish to host the application locally.

## Availability and requirements

**Project Name:** GENAVi.

**Project home page:** https://junkdnalab.shinyapps.io/GENAVi/

**Operating Systems:** Platform independent.

**Programming language:** R.

**Other requirements:** internet connection, internet browser.

**License:** GNU GPL version 3.

**Any restrictions to use by non-academics:** Not applicable.

## Additional file

Additional file 1: **Table S1.** List of cell lines used to generate RNA-Seq data to provide a resource for breast and ovarian cancer research. (XLSX 10 kb)

Additional file 2: **Table S2.** A featureCounts matrix of breast and ovarian cancer cell line panel. (XLSX 8428 kb)

Additional file 3: **Table S3.** A metadata table describing the samples in breast and ovarian cancer cell line panel to be used for DEA within GENAVi. (XLSX 9 kb)

Additional file 4: **Text 1.** Cell Culture Methods, RNA Isolation, Library Preparation, and Sequencing. **Text 2.** GENAVi User Guide. (DOCX 9534 kb)

Additional file 5: **Table S4.** A featureCounts matrix of CCLE ovarian cancer cell lines. (XLSX 14556 kb)

Additional file 6: **Table S5.** A metadata table describing CCLE ovarian cancer cell lines. (XLSX 9 kb)

## Data Availability

The RNA-Seq dataset generated in during the current study is available as fastq and raw expression counts at GEO; GSE114332. The gene expression count matrix is available online as Additional file 2: Table S2 for download via the app or within our publicly available data repository at .

## Abbreviations

DEA: Differential expression analysis
GUI: Graphical user interface
NGS: Next generation sequencing
