# Supplementary material for: GENAVi: a shiny web application for gene expression normalization, analysis and visualization
Source: BMC Genomics. 2019 Oct 16;20:745. doi: 10.1186/s12864-019-6073-7 (PMC6796420; doi:10.1186/s12864-019-6073-7)
Supplement: Supplementary file 4 — Text 1. Cell Culture Methods, RNA Isolation, Library Preparation, and Sequencing. Text 2. GENAVi User Guide. (DOCX 9534 kb) [file 12864_2019_6073_MOESM4_ESM.docx]

**Additional file 4: Text 1**

*Cell culture methods*.

We maintained all cell lines according to the recommended conditions (ATCC, VA). In house generated cell lines (IOSE and FTSEC) cell lines were grown in DMEM media, IOSE were supplemented with 10% FBS and FTSEC were supplemented with 2% Ultroser G (Pall Corporation). Cells were thawed and allowed to recover for 1 day. All cell lines were passaged and plated into 150mm plates. Cells were harvested at ~80% confluency and spun down, pellets were suspended and stored in Buffer RLT or QIAzol reagent (Qiagen, MD). A table of cell lines used for generating the resource is available as Supplementary Table 1. The cell line HeyA8 was used and labelled throughout as Hey.

*RNA isolation, library preparation and sequencing.*

Total RNA was isolated using RNeasy Mini Kit (Qiagen, MD) as per manufacturer’s instructions. RNA concentration and quality were determined by Qubit (ThermoFisher, CA) and Agilent 2100 using the RNA 6000 Nano kit (Agilent, CA). Total RNA were used as template for cDNA libraries and were prepared as poly-A selected libraries using the TruSeq v2 protocol (Illumina, San Diego, CA). Each library was pooled and the pool was sequenced across 4 lanes on the Illumina NextSeq500 in 2 × 75bp format generating an average of 33.4M reads.

*QC and Alignment of Sequencing*.

To assess the quality of our cell line data, we used a custom quality control script that calls FastQC (version 0.11.5) and fastqscreen (version 0.6.3) to provide input for MultiQC (version 1.3). Forty two cell lines from the CCLE related to ovarian cancer were downloaded as .bam files and converted to fastq using Picard SamToFastq. After review of QC metrics we aligned each read pair using a custom STAR script (version 2.5.1b) to Gencode v26 (hg38 build of the reference human genome). This process produced a bam file for each of our 20 cell lines which we then used for quantification of gene-level expression. The complete code for these scripts is available as an installable package at <https://github.com/alpreyes/GENAVi>.

*Generating Feature Counts*.

We used the featureCounts function of the subread package (version 1.5.2) to count the number of reads that mapped to a reference gene. Our use of the featureCounts function along with the Gencode v26 annotation file accounted for alternate transcripts of each gene and collapsed those transcripts to gene level. The resulting data table (Supplementary Table 2) had information on the quantified expression of 58219 features across our 20 cell lines and also included gene identification information, chromosomal position, strand information, and gene length. This data frame served as the foundation for assembling GENAVi.

*Data Availability.*

RNA-Seq data is available as fastq and raw expression counts at GEO (GSE114332). The gene expression count matrices are available as Supplementary Table 2, Supplementary 4, within the public Google Drive folder (<https://drive.google.com/open?id=1FocRZ6x05_0TwC_xiSIYwhsj9KAYNZwZ>) and vignette, from the GitHub repository (<https://github.com/alpreyes/GENAVi>) or for download via the app.

**Additional file 4: Text 2. GENAVi User Guide and Quick Start Tutorial**

# **GENAVi**

A Shiny Web Application for **G**ene **E**xpression **N**ormalization **A**nalysis and **V**isualization.

**Table of Contents**

1. **Introduction**
2. **Transformation and Normalization of Gene Expression Data**
3. **Differential Expression Analysis**
4. **Enrichment Analysis**
5. **Tutorial**

## **Introduction**

In order to improve access to normalization, visualization and analysis of RNA-Seq data we have combined a number of R packages using the Shiny web application format to create **GENAVi:** **G**ene **E**xpression **N**ormalization **A**nalysis and **Vi**sualization. This application allows users to browse the provided dataset; a panel of 20 cell lines commonly used in breast and ovarian cancer research, or upload their own dataset.

GENAVi is separated into three tabs:

**Gene Expression**: Users can search or browse a table of provided data or upload their own data, and apply different normalization methods. Genes can be found using the search bar, and then selected in the table or a list of gene symbols can be uploaded and automatically selected. The genes and normalization method selected on this tab will be retained for plotting and cluster analysis.

**Visualization:** Users can select between plotting a histogram of a single gene that has been selected in the data table (Expression plot), or heatmaps (Cluster or Correlation plots).

**Differential Expression Analysis:** Users can upload a metadata file providing information on sample groups to be compared, run differential expression analysis based on the DESeq2 package from R (PMID: 25516281), and plot the resulting P values in a customizable volcano plot.

## **Uploading User Data**

The Data Upload option of the app allows upload of a count matrix (ie. output from featureCounts) as a .csv file. Large matrices can take several minutes to upload and be normalized. A progress bar on the bottom right of the app screen tracks the task to completion. Once uploaded, user data replaces the provided data table. Gene names (as shown in column 1 of the data table) can be entered in the search field at the top right of the data table. A gene can be selected anywhere within the displayed row. Alternatively, the user can upload a list of gene symbols as a .txt file. Once the input gene list is uploaded, the corresponding genes within the displayed data table are automatically selected and can be used in the visualization tab of the application. Selected genes are automatically highlighted and moved to the top of the data table. The user can download the data table containing the selected genes with the “Download” option in the top left of the data table. The displayed data table can be sorted by any of the columns using the bi-directional arrows next to the column title.

## **Format of input count matrix**

We provide two examples of count matrices in a public google drive folder [*https://drive.google.com/drive/folders/1FocRZ6x05_0TwC_xiSIYwhsj9KAYNZwZ?usp=sharing*](https://drive.google.com/drive/folders/1FocRZ6x05_0TwC_xiSIYwhsj9KAYNZwZ?usp=sharing)

for users. The default data table within the app contains RNA-Seq data from a panel of 20 breast and ovarian cancer cell lines, however the user can also upload their own human or mouse RNA-Seq count matrix. Once a user uploads their own matrix the app uses the addgeneinfo() function to read in the count matrix in csv format. It checks if the first column is an ENSEMBLE ID or gene name. If the first column of the input count matrix is the proper format, addgeneinfo() adds gene metadata from Gencode hg38 for human data and mm19 for mouse data). If the first column of the user data count matrix is not an ENSEMBLE ID (ENSG IS) this can easily be added using BioMart (<https://biomart.genenames.org/martform/#!/default/HGNC?datasets=hgnc_gene_mart>). By uploading other forms of identifier, users can convert to ENSG ID by selecting “Ensemble gene ID” under the “Attributes>Gene Resources” menu at this page.

GENAVi filters out genes/features with zero reads mapped across all samples. This is applied to both the default data table and the user-uploaded matrix. This filtering step increases the speed of calculation for each transformation method described in the next section. By using GENAVi through the shiny server, the user can upload and process a count matrix up to approximately 15 Mb (representing ~100 samples) in 20 minutes. By hosting GENAVi locally on a more powerful server through the docker image or runGitHub() command, the user can increase the maximum upload size for a count matrix.

## **Transformation and Normalization of Gene Expression Data**

Variability in RNA-seq count data usually increases with expression level, indicating that genes that are more highly expressed can have a greater variation; this type of data is called heteroskedastic. Gene expression data in a raw count format causes cluster analysis such as PCA, hierarchical clustering, or k-means analysis to be driven by genes or features with the highest expression in the form of high raw count values. This is because in its raw count format, these highly expressed genes/features are also the most variable. To improve cluster analysis we need to convert this expression data to a homoskedastic form wherein genes/features have the same variance even for a higher range of the mean. To impose homoskedasticity on raw count data, historically the log2 transformation (log2(count + pseudocount)) has been used. While this method does prevent the most highly expressed genes/features from overpowering cluster analysis it can also give undue weight to the genes/features with the lowest counts depending on the choice of pseudocount. As an example; with the choice of 1 as the pseudocount in the case of log2 transforming a count value of 2, the resulting expression value is 1.584. Whereas with the same choice of pseudocount in the case of log2 transforming a count value of 200, the resulting expression value is 7.651. In this comparison, a difference in raw count expression that is two orders of magnitude is compressed to a difference of 6.067 under the log2 transform. It is relevant to understand the implementation as well as the drawbacks of this method as it used as the foundation for two of the transformations available in GENAVi; vst and rlog.

Another problem that arises with RNA-seq count data is overdispersion- the phenomenon wherein the observed variance within a dataset is greater than the expected variance under a chosen statistical model. Previously, the Poisson distribution has been used to model RNA-seq count data. However, a property of the Poisson distribution is that the mean and variance are equal; which prevents the model from accurately modeling RNA-seq count data, which is often heteroskedastic. A more appropriate statistical model is the negative binomial model (used in the edgeR package (PMID: [19910308](https://www.ncbi.nlm.nih.gov/pubmed/19910308)) and the DESeq2 package (PMID: 25516281)). This is an extension of the Poisson distribution that has different parameters allowing for separate modeling of mean and variance. Both edgeR and DESeq2 assume that the expected value of counts for a gene in a given sample can be modeled using this distribution.

We have provided four options for data normalization, which are described below. The user can toggle between transformation options by choosing from the “Select Transform” dropdown menu on left of the screen. Any choice of transformation will affect only the visualization and clustering of the displayed data. Differential expression analysis within GENAVi is performed on raw count data only as specified in the DESeq2 package.

### raw counts

The default version of expression data displayed in GENAVi is raw count data. This is the measure of expression produced by featurecounts. These raw count measures are integer values counting the number of reads mapped to a feature in the reference genome to which fastq files were aligned in a specific sample. Raw count data must be normalized to account for differences in sequencing depth between samples so that the user can make meaningful comparisons of gene expression levels across different samples.

### Row normalized

The “row normalized” transformation option is applied to raw count data similar to a Z-score normalization. For each gene in the displayed dataset, the mean expression and standard deviation across all samples/columns are calculated. The raw count expression values for each gene/feature are then transformed by subtracting the mean, and scaling by the standard deviation across all samples. A custom function was implemented to perform row normalization on each gene of the displayed data table. When opening GENAVi and/or uploading your own count matrix, the application acquires metadata matching the featurecounts table, calculates the row normalized transformation and saves it as an object to avoid calculating it each time the “row normalized” option is selected. This row normalization rescales the displayed data so that the gene expression is reported in units of standard deviation from the mean expression.

###

### Log-counts-per-million: logCPM

LogCPM transformation is implemented in GENAVi through the cpm() function from the edgeR package. This transformation is calculated in two steps. First, counts per million reads are calculated from raw counts by scaling the number of reads overlapping a feature in a specific sample by the total number of reads in that sample (library size). This is performed for every feature in every sample. These CPM values are then log2 transformed with the log=TRUE argument in the cpm() function.

When opening GENAVi and/or uploading your own count matrix, the application acquires metadata matching the featurecounts table (default or uploaded) and calculates the logCPM transform and saves it as an object to avoid calculating it each time the option is selected (implemented in the cpm() function in edgeR).

The logCPM transformation allows the comparison of gene expression across separate samples by accounting for differences in sequencing depth which allows for meaningful comparisons of gene expression across samples by accounting for between-sample variability.

### Variance stabilizing transformation- VST

The variance stabilizing transformation (VST), implemented in the DESeq2 package, attempts to address the issue of overdispersion of RNA-seq count data. VST transformation produces transformed expression measures similar to those produced by the log2 transform when used on genes with high counts. Similar to the edgeR package, the VST transformation also assumes a negative binomial model to allow for a change in variance based on the dynamic range of the mean. When clustering or analysis is being performed on a genome-wide dataset (all genes), VST transformed counts approach homoskedasticity and can be used directly for clustering.

VST is much faster to compute than rlog and is less sensitive to counts with high values. Thus, we recommend VST transformation in larger datasets (up to hundreds of samples/columns), rather than rlog. When opening GENAVi and/or uploading your own count matrix, the application acquires metadata matching the featurecounts table (default or uploaded) and calculates the vst transform and saves it as an object to avoid calculating it each time the option is selected (implemented in the rlog function from DESeq2)

###

### Regularized-logarithm transformation- rlog

GENAVi uses the regularized logarithm (rlog) transform implemented in DESeq2, which performs similarly to the log2 transformation when applied to genes/features with high raw count expression. When rlog is applied to lower count values, the resulting transformed values are more shrunken towards the mean value of a gene’s expression across all samples in the data. Rlog transformed values are calculated by converting the raw count data to the log2 scale and then defining a size factor for each sample which accounts for differences in sequencing depth between samples. This approach is referred to as shrinkage. rlog transformed counts approach homoskedasticity, and can be used directly for clustering. The calculation of rlog in large datasets is more computationally intensive, so users with a large number of samples/columns in their featureCounts matrix should note that this step of data upload requires several minutes.

**Bringing Previously Normalized Data to GENAVi.**

Users who have only previously normalized gene expression data available (ie. data in a .tpm (transcripts per million), RPKM (Reads per Kilobase Million), FPKM (Fragments per Kilobase Million format) can also use GENAVi. Although the normalization methods and differential expression analysis cannot be performed on previously normalized data, the data visualization modules can be used for data selection, clustering and plotting. Users uploading previously normalized data should ensure the ‘Select Transform’ menu on the ‘Gene Expression’ tab of the app be set to ‘raw counts’ to ensure their data isn’t further normalized.

##

## **Data Visualization**

### Barplot

Expression of a single gene/feature across all samples can be displayed in a simple barplot. The data format selected from the Gene Expression tab (ie. normalized or raw) for a single gene selected in the data table will be plotted. If more than one gene is selected from the data table, the simple barplot in the Visualization tab is disabled and an interactive heatmap is displayed instead.

*Figure 1. Gene expression across samples can be easily visualized using the Expression barplot.*

*
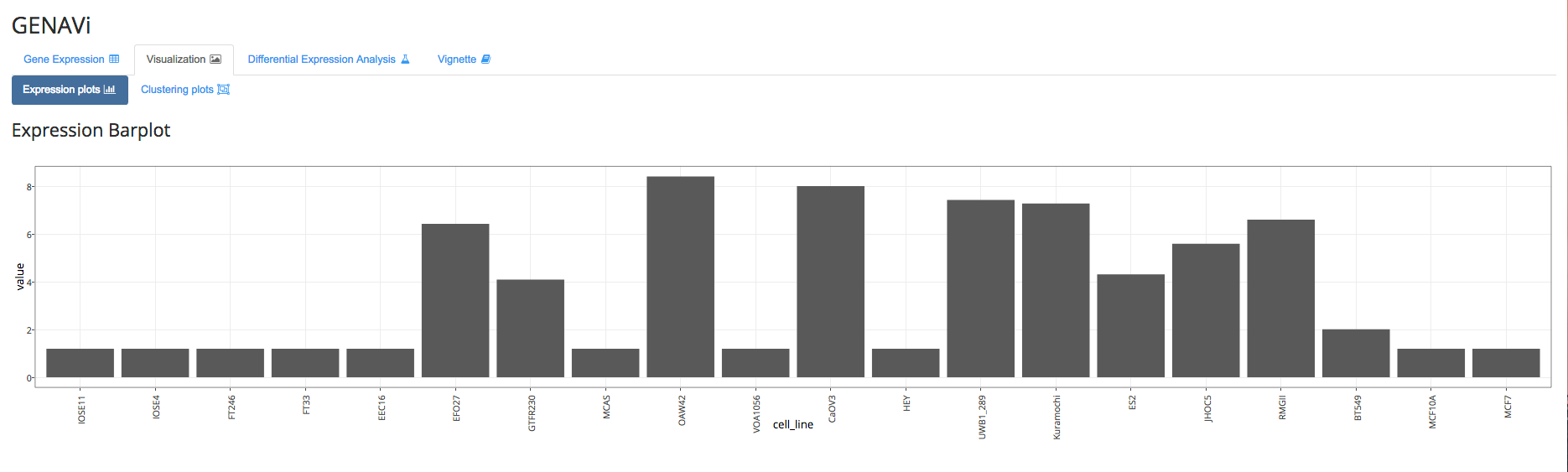
*

###

### Expression heatmap

GENAVi uses the R package iheatmapr to generate interactive heatmaps to visualize gene expression information. All capabilities of manipulating these heatmaps that iheatmapr provides are available in GENAVi. The user can hover over cells in the displayed heat map to see row and column identification information, subset the heatmap to zoom in on cells of interest, and download the heatmap by clicking on the camera icon in the top right corner.

To generate a gene expression heatmap, the user first selects the appropriate normalized data table by selecting from the “Select Transform” dropdown menu on the left side of the application. Normalization strategies for different applications is described above. It is worth noting that visualizing the “raw counts” version of an RNA-seq data set may make the heatmap color scale difficult to interpret because of the magnitude of the range of gene expression in raw counts. Next, the user can select genes/features (either by searching for and clicking on individual genes or uploading a gene list test file). Once the desired genes/features are selected in the displayed data table, the user can navigate to the Visualization tab and select the “Expression plots” subtab to see the resulting expression heatmap.

The expression heatmap displays the expression matrix of the user-selected genes across all samples of the displayed dataset under the selected version of the data table with a dendrogram/hierarchical clustering of samples. The user may see that the order of the samples (columns) and selected genes/features in the expression heatmap is not the same as the order in the displayed data table or the order of selection. This is due to the add_col_dendro() and add_row_dendro() options in the iheatmapr package. These options order the columns and rows of the expression heatmap by similarity of expression of the selected genes/rows. This similarity is measured by calculating the Euclidean distance between the samples of the gene expression matrix by applying the dist() function directly to the gene expression information.

*Figure 2. Expression data can be visualized in a clustered heatmap.*

**
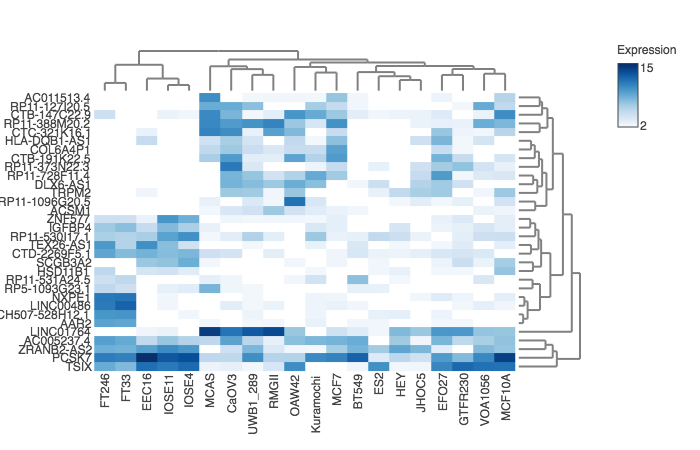
**

### Correlation heatmap (Clustering heatmap)

In addition to viewing the expression matrix of selected genes, the user can also view how the expression of selected genes affects the similarity between samples measured through correlation. By selecting “Clustering plots” subtab, the user can view a heatmap representing the the Pearson correlation matrix calculated from the gene expression matrix. The correlation matrix is calculated using the cor() function with the option method=”pearson” option which produces a matrix containing the pairwise pearson correlations between the columns of the displayed data table. When viewing the correlation heatmap, the user can select to either cluster samples based on the entire gene expression data set by selecting “All genes” from the dropdown menu in the top left of the tab. The user can also choose “Selected genes” to cluster samples using the gene expression information from only the selected genes/features. The order of the columns or rows may not be maintained from the data table due to the clustering applied using the add_col_dendro() and add_row_dendro() options in the iheatmapr package. Unlike the expression heatmap where these options order columns and rows based on the Euclidean distance calculated directly from the gene expression matrix, the correlation heatmap orders columns and rows based on a matrix of Pearson distance computed by subtracting each pairwise correlation coefficient from one. The user can also use the “Cluster correlation” dropdown menu to toggle between viewing sample correlation across specified genes or gene correlation across samples.

*Figure 3. Correlation between samples can be visualized using the Cluster heatmap function.*

*
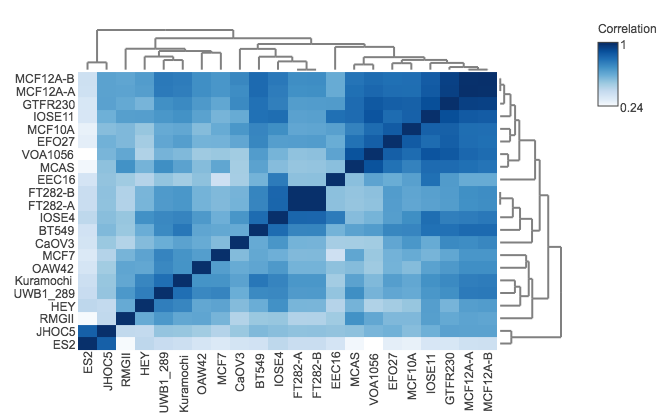
*

##

## **Differential Expression Analysis**

### Background

A natural use/application of RNA-seq data is differential expression analysis (DEA): the use of statistical methods to quantify the effects of experimental conditions on gene expression. To do this, GENAVi utilizes the R package DESeq2. The differential expression analysis functions within DESeq2 use negative binomial generalized linear models as well as empirically driven prior distributions to model gene count distributions, estimate dispersion, and calculate log fold changes. GENAVi performs these DEA functions only on raw count data, so any choice of transformation does not affect the functions in this tab.

The user can define experimental conditions of their uploaded data, perform differential expression analysis, define statistical thresholds, and visualize results within GENAVi.

To perform differential expression analysis with the provided count matrix and accompanying metadata table, the user can download the example metadata file directly from within GENAVi in the “Differential Expression Analysis” tab. After uploading this file to the DEA tab, the user will see each sample in the default count matrix with accompanying information in the “model” and “batch” columns. By selecting “model” as the condition column, “batch” as the covariate, and “precursor_normal” as the reference level, the user can run the DEA pipeline comparing different subtypes of Ovarian Cancer to our selected normal cell line group. These steps will allow the user to recreate our DEA results table from which a list of top differentially expressed genes can be extracted. By uploading this list of differentially expressed genes to GENAVi, the user can recreate our heatmaps.

###

### Uploading metadata and defining a model for DEA

Before performing differential expression analysis, the user must first upload a metadata file, in csv format, containing the experimental conditions of each sample in the displayed data. This is done by clicking the “Browse...” button in the “Metadata upload” section of the tab. The first column of the csv file should be labeled “sample” and each row of that column should be an exact column name of the uploaded counts matrix. The following columns can be labeled in whatever manner reflects the experimental design conditions and batching used to generate the samples. We have included an example metadata csv file that describes the grouping of the samples in our cell line panel as well as a “batch” column that reflects how the samples were prepared for sequencing.

After the metadata table has been uploaded, the user can then select the variables to be used for differential expression analysis. The user can select the experimental condition of interest from the “Select condition column for DEA” drop down menu that contains the information from the metadata file. Next the user can select a covariate to correct for under the “Select covariates for DEA” drop down menu. We recommend that sequencing or library preparation batches can be included in this column, and that this covariate account for batch effect between samples should they be present. Lastly, the user must select the reference condition of the selected experimental condition (the group that each group will be compared to). This is done by selecting from the “Select reference level for DEA” dropdown menu which will again contain information from the uploaded metadata table. The gene expression information within this reference group will be used as the baseline for comparisons with all other samples. For example, in the case where the selected experimental condition reflected drug treatment, the reference level would be the untreated group.

Once all parts of the model are defined, the user can click “Perform DEA”, and then GENAVi will produce the results of differential expression analysis by implementing the DESeq() function. This analysis may take some time and a progress bar reflecting the current stage of analysis will be displayed in the bottom right of the screen.

### DEA results tables

After performing differential expression analysis, a table of results for each comparison between the experimental condition to the reference condition will be displayed. The results table for each pairwise comparison can be selected using the “DEA - select results” dropdown menu. The results of the differential expression analysis will be shown in a table giving base mean, log2 fold changes, standard errors, test statistic, p-value, and adjusted p-value (Benjamini-Hochberg adjustment).

The columns in the table provide:

- **baseMean** reports the average value of normalized counts for individual genes across all samples in the displayed data table.
- **log2FoldChange** is an estimate of the effect size of expression changes caused by experimental conditions relative to the specified reference level for individual genes.
- **lfcSE** reports the uncertainty around the log2 fold change estimate as it is computed using maximum likelihood.
- **stat** reports the output of the Wald test that each gene undergoes which serves as another estimate of effect size.
- **p value** reports the probability of observing an effect size equal to or greater than the observed if the null hypothesis were true.
- **padj** reports the adjusted p value computed using the Benjamini-Hochberg (BH) adjustment. Adjusting the raw p value is necessary for high-throughput data to account for multiple testing and to minimize reporting of false positives. The volcano plot (described in the following section) displays results using this adjusted p value rather than the raw p value.

### Volcano Plot

The DEA results can be visualized in an interactive volcano plot under the “Volcano plot” subtab. In this plot, the y-axis displays the -log10(p-value adjusted) and the x-axis displays the log2FoldChange for individual genes. This type of plot is commonly used to identify which genes are downregulated, upregulated, or unchanged given the experimental conditions. Within the “Volcano plot” menu, the user can specify threshold values for both axes which are displayed on the volcano plot as dashed vertical and horizontal lines and can be used to categorize genes based on changes in their expression. Genes that fall into these different categories are colored on the plot accordingly. Additionally, the user can see more information about individual genes by hovering over and clicking on individual points. We recommend that the user check the “Perform Log fold change shrinkage” box under the “DEA - Select Results” menu before viewing the volcano plot. This is because genes with low counts tend to have higher variability in the log fold change estimates. This phenomenon is closely related to the heteroskedasticity of count data because the difference in variability across samples for lowly and highly expressed genes complicates comparisons of effect size. If this option is selected, GENAVi uses the apeglm method to shrink LFC estimates of genes toward zero based on mean expression across samples as well as dispersion.

## **Enrichment Analysis**

### Background

An important step after identifying differentially expressed genes is to retrieve a functional profile of those genes to understand the underlying biological processes represented in the original data. Several databases provide annotated gene sets that can be used for this type of analysis (i.e. Molecular Signatures Database, and Kyoto Encyclopedia of Genes and Genomes). GENAVi utilizes the clusterProfiler package to provide accessibility to these and other tools to investigate enriched gene sets represented by the displayed data.

**Performing Enrichment Analysis**

To utilize the functions in GENAVi’s “Enrichment analysis” tab, the user must first upload a DEA results file (in csv format). This is done by clicking the “Browse...” button in the “DEA results upload” section within this tab. The user can also download an example DEA results file in this tab. We provide a subset of TCGA breast cancer data, accessed via TCGAbiolinks, and retained only samples with luminal or basal subtype from cases of African American ancestry to generate a modestly sized test data set for users. The code used to collect this data is available at the GitHub repository for GENAVi (<https://github.com/alpreyes/GENAVi>). Once a DEA results file is loaded, the user can select between “Upregulated” and “Downregulated” in the “Gene status” dropdown menu to specify the subset of genes to be used for an Over Representation Analysis (ORA). This process determines what gene sets are enriched based on the status of differentially expressed genes. The results of this ORA can be visualized as either dot plot or enrichment map under the “Plot type” dropdown menu. A thorough tutorial for this suite of analysis tools is available at <https://guangchuangyu.github.io/pathway-analysis-workshop/>.

Using this selected subset of genes, the user can also choose to perform Gene Set Enrichment Analysis (GSEA) which performs a similar function to ORA but does not look exclusively at differentially expressed genes. For both ORA and GSEA, the user must first specify a database using the “Select the type of analysis” dropdown menu. Lastly, under the “Plot type” dropdown menu, the user can now select between five different types of visualization that report the results of this analysis.

*Figure 4. Example of Volcano plot to visualize differential expression analysis.*


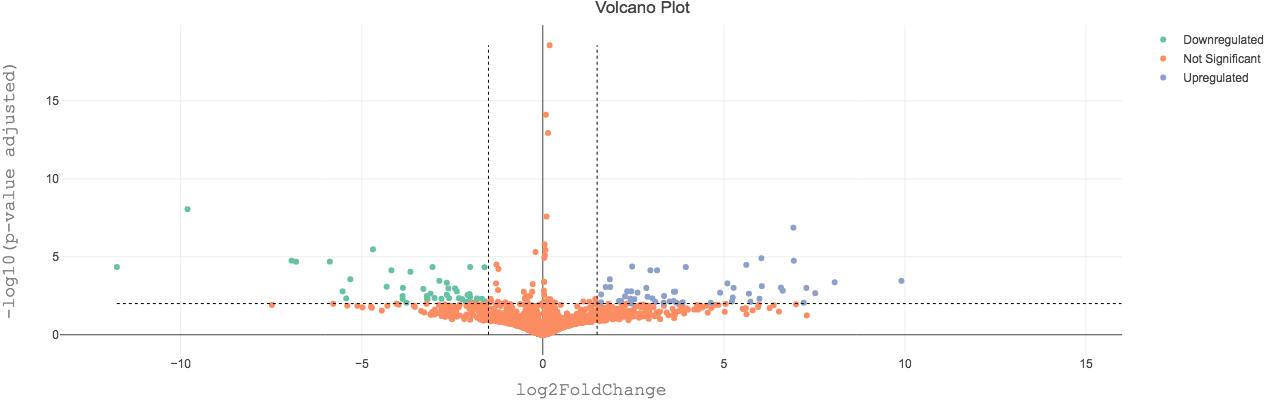


###

### Installing GENAVi to run locally.

We recommend datasets larger than 100 samples be run on a local machine with >8GB of RAM. In order to support this we have created a docker image containing the application and the R Shiny environment, which can be downloaded at: [https://hub.docker.com/r/cedarscompbio/genavi/](file:///Users/jonesmrx/Box%20Sync/OvCa/RNASeq_Cell_Lines/GENAVi%20Manuscript/via%20a%20docker%20image%20through%20this%20link:%20https:/hub.docker.com/r/cedarscompbio/genavi). Additionally, GENAVi can be run on a users local machine or server by entering the command: >shiny::runGitHub("alpreyes/GENAVi") in an RStudio window. This will install all necessary R packages, all source code used to build GENAVi will be downloaded directly from the GitHub repository, and the application will be hosted and run locally.

# **Tutorial**

This quick-start tutorial will guide you through using the provided data files to recreate a normalization, plotting and differential expression analysis.

A set of example files for use with this tutorial can be found at;

<https://drive.google.com/drive/folders/1FocRZ6x05_0TwC_xiSIYwhsj9KAYNZwZ?usp=sharing>

Download and save the following files;

GENAVi_Cell_Line_RNA_seq_count_matrix.csv

GENAVi_Cell_Line_RNA_seq_metadata.csv

GENAVi_gene_list_input.txt

Open junkdnalab.shinyapps.io/GENAVi/

In the ‘Gene Expression’ tab of GENAVi, select the ‘Data upload’ button to bring up a separate menu then select the ‘Browse’ button to upload your count matrix.

Select the ‘Visualization’ tab, and then the ‘Clustering Plots’ sub tab. By default, Figure 3 from the Vignette will be created, showing the clustering of all samples by all genes shown in the data table.

To plot the expression of individual genes as bar charts select the appropriate normalization method (a detailed explanation of which normalization method is suited to different datatypes is available in the Vignette). Variance stabilization transformation is optimal for most datasets for this purpose. Search and select the gene of interest in the data table. Once selected, move to the ‘Visualization’ tab then the ‘Expression plots’ sub-tab.

To perform DEA select the ‘Differential Expression Analysis’ tab. Select the ‘Metadata upload’ to bring up a separate menu then select the ‘Browse’ button to upload the metadata file, which will be displayed in the app.

The settings for DEA can then be set by selecting the ‘DE analysis’ button which will bring up a separate menu. The column headers of the metadata file will be listed under the ‘condition’ and ‘covariate’ menus for selection. In this example select ‘model’ as the condition and ‘batch’ as the covariate to perform DEA between different Ovarian Cancer subtypes correcting for sequencing batch.

­­The DEA results are displayed in the main pane. A full description of each column in the table is available in the Vignette.

A volcano plot can be generated with customized parameters for fold change and P value thresholds. First, we recommend performing log fold change shrinkage on the DEA results by clicking the ‘Select Results’ button then selecting the checkbox next to ‘Perform Log fold change shrinkage’. To recreate Figure 4 from the Vignette, select the ‘Volcano plot’ button to bring up a separate menu then set a log2FoldChange threshold of 1.5 and a P adjusted cut off of 0.01.

The results of DEA can be downloaded through the pop-up menu that appears after clicking the ‘Select Results’ button then selecting the ‘Download DEA Results’ button. These can easily be used to generate gene lists. An example gene list labeled GENAVi_gene_list_input.txt is in the Google Drive folder and can be used to select differentially expressed genes to include in clustering or expression plots (such as Figure 2 in the Vignette).

You can use the DEA results as input for enrichment analysis of gene expression data with regard to functional gene sets and pathways. For more information, please read this workshop material <https://guangchuangyu.github.io/pathway-analysis-workshop>. A video demonstrating this part of the tool is available at <https://www.youtube.com/watch?v=EJowR5ScARE&feature=youtu.be>

- Among the options, GENAVi offers:
  - Gene Set Enrichment Analysis (GSEA) (Subramanian et al. 2005)
  - Over Representation Analysis (ORA) (Boyle et al. 2004)
- For GSEA, GENAVi provides the following ranking methods:
  - logFC
  - -log10(Pvalue) / sig(logFC)
  - -log10(Pvalue) * logFC
- GENAVi provides the following analysis methods:
  - WikiPathways analysis
  - MSigDB analysis
  - Gene Ontology analysis
  - KEGG analysis

To obtain the code used to perform analysis and generate plots in GENAVi, select the ‘Generate report’ button in any tab to download an html file outlining the analysis performed in that section.
